# Quantitative measures of estrogen receptor expression in relation to breast cancer-specific mortality risk among white women and black women

**DOI:** 10.1186/bcr3486

**Published:** 2013-09-27

**Authors:** Huiyan Ma, Yani Lu, Polly A Marchbanks, Suzanne G Folger, Brian L Strom, Jill A McDonald, Michael S Simon, Linda K Weiss, Kathleen E Malone, Ronald T Burkman, Jane Sullivan-Halley, Dennis M Deapen, Michael F Press, Leslie Bernstein

**Affiliations:** 1 Division of Cancer Etiology, Department of Population Sciences, Beckman Research Institute, City of Hope, Duarte, CA 91010, USA; 2 Division of Reproductive Health, Centers for Disease Control and Prevention, Atlanta, GA 30333, USA; 3 Center for Clinical Epidemiology and Biostatistics, Department of Biostatistics and Epidemiology, University of Pennsylvania School of Medicine, Philadelphia, PA 19104, USA; 4 Karmanos Cancer Institute, Department of Oncology, Wayne State University, Detroit, MI 48201, USA; 5 Cancer Centers Branch, National Cancer Institute, Bethesda, MD 20892, USA; 6 Division of Public Health Sciences, Fred Hutchinson Cancer Research Center, Seattle, WA 98109, USA; 7 Department of Obstetrics and Gynecology, Baystate Medical Center, Springfield, MA 01199, USA; 8 Department of Preventive Medicine, University of Southern California, Los Angeles, CA 90033, USA; 9 Department of Pathology, Keck School of Medicine, University of Southern California, Los Angeles, CA 90033, USA

## Abstract

**Introduction:**

The association of breast cancer patients’ mortality with estrogen receptor (ER) status (ER + versus ER-) has been well studied. However, little attention has been paid to the relationship between the quantitative measures of ER expression and mortality.

**Methods:**

We evaluated the association between semi-quantitative, immunohistochemical staining of ER in formalin-fixed paraffin-embedded breast carcinomas and breast cancer-specific mortality risk in an observational cohort of invasive breast cancer in 681 white women and 523 black women ages 35-64 years at first diagnosis of invasive breast cancer, who were followed for a median of 10 years. The quantitative measures of ER examined here included the percentage of tumor cell nuclei positively stained for ER, ER Histo (H)-score, and a score based on an adaptation of an equation presented by Cuzick and colleagues, which combines weighted values of ER H-score, percentage of tumor cell nuclei positively stained for the progesterone receptor (PR) and human epidermal growth factor receptor 2 (HER2) results. This is referred to as the ER/PR/HER2 score.

**Results:**

After controlling for age at diagnosis, race, study site, tumor stage, and histologic grade in multivariable Cox proportional hazards regression models, both percentage of tumor cell nuclei positively stained for ER (*P*_trend_ = 0.0003) and the ER H-score (*P*_trend_ = 0.0004) were inversely associated with breast cancer-specific mortality risk. The ER/PR/HER2 score was positively associated with breast cancer-specific mortality risk in women with ER + tumor (*P*_trend_ = 0.001). Analyses by race revealed that ER positivity was associated with reduced risk of breast cancer-specific mortality in white women and black women. The two quantitative measures for ER alone provided additional discrimination in breast cancer-specific mortality risk only among white women with ER + tumors (both *P*_trend_ ≤ 0.01) while the ER/PR/HER2 score provided additional discrimination for both white women (*P*_trend_ = 0.01) and black women (*P*_trend_ = 0.03) with ER + tumors.

**Conclusions:**

Our data support quantitative immunohistochemical measures of ER, especially the ER/PR/HER2 score, as a more precise predictor for breast cancer-specific mortality risk than a simple determination of ER positivity.

## Introduction

The estrogen receptor (ER), which was identified in the late 1960s, is a protein molecule located in the nuclei of hormone target cells [1]. ER contains a specific ligand binding domain to which only estrogen or closely related molecules can bind. The positivity of ER in breast cancer tissue was first considered a strong indicator of response to endocrine therapy in the early 1970s [2] and was first recognized as a prognostic factor in the late 1970s [3].

Historically, ER expression in breast tissue was quantified using ligand binding assays, such as the most commonly used dextran-coated charcoal (DCC) assay [4,5]. Ligand binding assays quantify the amount of ERs with unoccupied ligand binding domains (those that have not bound estrogen or a closely related molecule) by measuring the amount of radiolabeled specific binding of estradiol in tissue homogenates [6]. Since the development of monoclonal antibodies to ER in the 1980s, ligand binding assays have been gradually replaced by monoclonal assays that measure both unoccupied and occupied ERs [7,8]. There are two types of monoclonal assays: the quantitative enzyme immunoassay [7] and the semi-quantitative immunohistochemistry (IHC) assay [8,9].

The ease of performing an IHC assay on routinely prepared formalin-fixed paraffin-embedded tissue blocks, combined with the assay’s ability to evaluate small tumor samples and to ensure that only tumor cells are assessed, has led to the IHC assay becoming the first choice for ER measurement in pathology. The lack of a standardized cutoff point for ER positivity has been a longstanding issue. However, in 2010 the joint panel of the American Society of Clinical Oncology and the College of American Pathologists published guidelines recommending that ≥1% of tumor nuclei positively stained for ER should be the cutoff point for ER positivity [10]. The panel also noted that few follow-up studies had been published assessing quantitative staining of ER in tissue as a prognostic indicator. In 2011 Cuzick and colleagues reported that, in the Arimidex, Tamoxifen, Alone or in Combination trial, the quantitative ER Histo (H)-score alone or in combination with three other markers (progesterone receptor (PR), human epidermal growth factor receptor 2 (HER2) and Ki-67) was associated with risk of distant recurrence in postmenopausal women who were diagnosed with ER-positive breast cancer [11].

Here we present results from an observational cohort study of white women and black women with invasive breast cancer in which two quantitative measures of ER alone and an adaptation of Cuzick’s combined score, based on quantitative values of ER and PR, and HER2 status [11], were assessed to determine how they compare with each other and with ER status (positive versus negative) in predicting mortality risk.

## Methods

### Study population and data collection

The participants were breast cancer patients from Detroit and Los Angeles (LA) who participated in the Women’s Contraceptive and Reproductive Experiences (CARE) Study, a population-based, case–control study designed to examine risk factors for invasive breast cancer among white women and black women [12]. The Women’s CARE Study selected a stratified (by age group) random sample of women ages 35 to 64 years who were newly diagnosed with histologically confirmed, first primary invasive breast cancer (International Classification of Diseases for Oncology codes C50.0 to C50.9) between July 1994 and April 1998. Black women were oversampled to maximize their numbers in the study, and white women were sampled to provide approximately equal numbers of women in each 5-year age group between 35 and 64 years. The Women’s CARE Study recruited and interviewed 1,921 breast cancer patients from Detroit (*n* = 679) and LA (*n* = 1,242). These two study sites were selected to collect tumor tissue samples based on representative case participants in the Women’s CARE Study and the ability to obtain tumor tissue samples. All participants provided written informed consent. The study protocol was approved by the Institutional Review Boards at the University of Southern California, the Karmanos Comprehensive Cancer Center, the Centers for Disease Control and Prevention, and the City of Hope.

### Assessment of biomarkers

Formalin-fixed paraffin-embedded tumor blocks were successfully retrieved from pathology laboratories where diagnoses were made for 1,333 participating breast cancer patients (Detroit, *n* = 414; LA, *n* = 919), which was approximately 80% of those requested at each site. Tumor blocks were not requested for all patients from these two sites due to financial constraints. All tumor blocks were carefully reviewed and evaluated in MFP’s pathology laboratory at University of Southern California.

We excluded 127 patients’ samples because the tumor blocks contained only carcinoma *in situ* (*n* = 56) or no tumor tissue (*n* = 46); had insufficient tissue for assay (*n* = 3); contained only hematoxylin-and-eosin-stained tissue (*n* = 8); or had other problems that made the evaluation difficult (*n* = 14). The expression of ER was determined for the remaining 1,206 samples (Detroit, *n* = 367; LA, *n* = 839).

The expression of ER was determined using previously published IHC methods [13]. In brief, the ER IHC method involved heat-induced antigen retrieval (pH 6.0) with a sodium citrate buffer, the use of a commercially available anti-ER mouse monoclonal primary antibody (1D5, 1:50 dilution; Zymed, Inc., South San Francisco, CA, USA), a biotinylated secondary rabbit anti-mouse antibody (Zymed, Inc.) with horseradish peroxidase-labeled streptavidin (Zymed, Inc.), and detection with diaminobenzidine. Immunostaining results for ER were scored semi-quantitatively on the basis of the visually estimated percentage of positively stained tumor cell nuclei. The intensity of nuclear staining was scored for individual tumor cell nuclei as negative (–)/no staining, staining weakly (+), staining intermediately (++), or staining strongly (+++). A minimum of 100 tumor cells were scored with the percentage of tumor cell nuclei in each category recorded. The sum of three staining categories equates to the overall percentage of positively stained tumor cell nuclei. If ≥1% of tumor cell nuclei stained positively, the tissue sample was considered ER-positive. The ER H-score was calculated as a weighted sum of the intensity of IHC tumor cell nuclei as follows [14,15]:

$$\begin{array}{c} \text{ER}\;H‒\text{score}=(\%\;\text{of}\;\text{positively}\;\text{stained}\;\text{tumor}\;\text{cell}\;\text{nuclei}\;\\ \;\text{at}\;\text{weak}\;\text{intensity}\;\text{category}\times 1)\\ \;+(\%\;\text{of}\;\text{positively}\;\text{stained}\;\text{tumor}\;\text{cell}\;\\ \;\text{nuclei}\;\text{at}\;\text{intermediate}\;\text{intensity}\;\text{category}\times 2)\\ \;+(\%\;\text{of}\;\text{positively}\;\text{stained}\;\text{tumor}\;\text{cell}\;\text{nuclei}\;\text{at}\;\\ \;\text{strong}\;\text{intensity}\;\text{category}\times 3) \end{array}$$

IHC for PR and HER2 was also conducted in the same central pathology laboratory using methods that have been described previously [16]. PR expression was quantified as the percentage of tumor cell nuclei positively stained for PR. For HER2, no (0) or weak (1+) membrane protein immunostaining was considered low HER2 expression (HER2–); and moderate (2+) or strong membrane protein immunostaining (3+) was considered HER2 overexpression (HER2+) based on previously validated results from the same pathology laboratory comparing IHC with *HER2* gene amplification measured by fluorescent *in situ* hybridization methods [17].

A combined score of the ER H-score with the percentage of tumor cell nuclei positively stained for PR and HER2 positivity was generated according to Cuzick’s IHC4 equation [11]:

$$\begin{array}{c} \mathrm{IHC}4=94.7\times [–0.100{\;\mathrm{ER}}_{10}–0.0{79\;\mathrm{PR}}_{10}+0.586\;\mathrm{HER}2\\ \;+0.240\;\ln\left(1+10\;\mathrm{Ki}67\right)] \end{array}$$

We did not assay Ki67 so excluded that part of the equation. The variable ER_10_ was generated by dividing the ER H-score by 30; PR_10_ was calculated as the percentage of tumor cell nuclei positively stained for PR divided by 10; HER2 was scored as 0 if negative and as 1 if positive. The combined score is referred to as the ER/PR/HER2 score in this article.

### Tumor characteristics

The Women’s CARE Study collected tumor stage, histologic grade, hormone receptor status, and other tumor characteristics from the Detroit and LA Surveillance Epidemiology and End Results (SEER) cancer registries. We previously reported that the agreement between the centralized pathology laboratory classification and reported ER status in the SEER registry was substantial (κ = 0.70) [18]. Two women were missing information on the tumor stage and were excluded from the statistical analyses; the final sample size for this analysis was 1,204 women (race: 681 white, 523 black; tumor stage: 697 localized, 507 nonlocalized).

### Vital status follow-up

Participants were followed annually for vital status, date of death and cause of death, using standard SEER follow-up procedures. Data on vital status for Detroit participants were available through 31 December 2004; patients in LA were followed through 31 December 2007.

### Statistical analysis

Analyses were conducted using deaths due to breast cancer (International Classification of Diseases codes ICD9-174, ICD10-C50) as endpoints. Follow-up in days began with breast cancer diagnosis and ended with the woman’s death due to breast cancer (*n* = 272), her death due to another cause (*n* = 63), or the end of the follow-up period (*n* = 869). We did not include deaths due to causes other than breast cancer as endpoints because we assumed that the quantity of ER expression would not be related to these deaths.

Multivariable Cox proportional hazards models were fit to the data to estimate the hazard ratio (HR) representing the relative risk of death due to breast cancer associated with an ER measure (percentage of tumor cell nuclei positively stained for ER, ER H-score, or the ER/PR/HER2 score) and the 95% confidence interval (CI) for the HR [19]. Models were fit to all data and by race (white, black). These models used age (in days) as the time metric, were stratified by age in years at diagnosis, and were adjusted for race, study site, and tumor stage (Model 1). A second analysis included additional adjustment for histologic grade (Model 2). The participants’ frequency distributions for each of the covariates included in these models overall and by race have been described elsewhere [20]. We categorized the percentage of tumor cell nuclei positively stained for ER as a five-category variable (<1%, 1 to 39%, 40 to 59%, 60 to 79%, 80 to 100%) and used previously published categories for the ER H-score [21]. We categorized the ER/PR/HER2 score for women diagnosed with ER-negative tumor and those with ER-positive tumor, separately. The combined score was categorized as a three-category variable (<0, 0, >0) in women with ER-negative tumors since approximately 67% of them scored 0. The combined score was categorized as a four-category variable according to quartile distribution of women with an ER-positive tumor. The association between the combined score and breast cancer-specific mortality risk was examined for women with ER-negative tumors and those with ER-positive tumors, respectively.

Tests for trend were conducted by fitting ordinal values corresponding to each category of the variable (percentage of tumor cell nuclei positively stained for ER, ER H-score, ER/PR/HER2 score) and determining whether the coefficient (slope of the dose response) differed statistically from 0. We also obtained HRs (95% CIs) associated with each 10% increase in tumor cell nuclei positively stained for ER and with each 20 unit increase in the ER H-score.

To test potential effect modification by race, we constructed a likelihood ratio test comparing two multivariable Cox proportional hazards models (likelihood ratio test for heterogeneity of HRs for dichotomous variable (ER-positive vs. ER-negative), HRs for trends, HRs for per 10% increase in tumor cell nuclei positively stained for ER, or HRs for per 20 unit increase in ER H-score, with 1 degree of freedom) [22].

All reported *P* values are two-sided. All statistical analyses were performed using SAS version 9.2 software (SAS Institute, Cary, NC, USA).

## Results

### Descriptive characteristics of ER expression overall and by follow-up outcome

**Figure 1:**
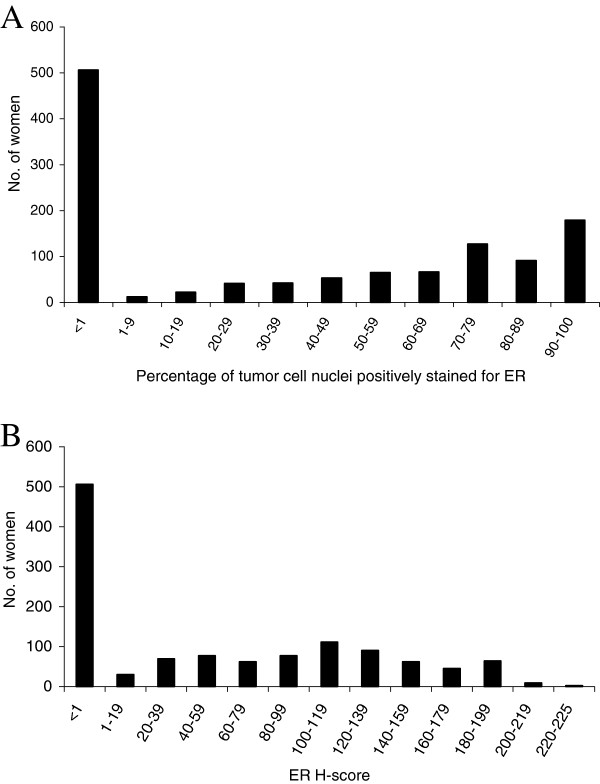
**Frequency distribution of estrogen receptor expression.** Percentage of tumor cell nuclei positively stained for estrogen receptor (ER) **(A)** and ER Histo (H)-score **(B)** in 1,204 white women and black women diagnosed with invasive breast cancer.

The frequency distributions of the percentage of tumor cell nuclei positively stained for ER and ER H-score are shown in Figure 1A and B, respectively.

Of 1,204 women, 58% were diagnosed with ER-positive (≥1% positively stained tumor cell nuclei) breast cancer (Table 1). The percentage of women with ER-positive breast cancer was lower among women who died from breast cancer (42.5%) than among women who died from other causes (67.0%) or among women who were alive at the end of the follow-up period (61.8%). The differences across these three outcome groups were also observed by race. Further, black women were less likely to have ER-positive disease than white women (overall: 51.3% vs. 62.5%).

**Table 1:** Descriptive statistics for the measures of estrogen receptor expression by follow-up outcome

	**Overall**	**Follow-up outcome**			
		**Died from breast cancer**	**Died from other causes**	**Alive at the end of follow-up**	***P***_**F-test**_
**Overall**	*n* = 1,204	*n* = 272	*n* = 63	*n* = 869	
ER-positive^a^ (%)	58.0	42.5^b^	67.0^b^	61.8^b^	
Among women with ER-positive^a^ tumor					
% of tumor cell nuclei positively stained for ER	64.7 (25.5)	62.2 (27.0)	65.6 (24.5)	65.1 (25.2)	0.53
ER H-score^c^	100.8 (53.0)	93.0 (52.9)	104.5 (54.6)	102.2 (52.9)	0.22
ER/PR/HER2 score^d^	–64.8 (40.6)	–49.9 (43.5)	–65.7 (34.7)	–67.9 (39.8)	<0.0001
**White women**	*n* = 681	*n* = 131	*n* = 24	*n* = 526	
ER-positive^a^ (%)	62.5^b^	47.2^b^	78.6^b^	65.8^b^	
Among women with ER-positive^a^ tumor					
% of tumor cell nuclei positively stained for ER	64.6 (25.9)	58.0 (27.4)	74.4 (18.1)	65.2 (25.7)	0.03
ER H-score^c^	100.7 (53.2)	82.3 (48.8)	122.1 (44.9)	102.8 (53.7)	0.004
ER/PR/HER2 score^d^	–67.6 (39.7)	–46.1 (44.7)	–78.8 (26.3)	–70.8 (38.2)	<0.0001
**Black women**	*n* = 523	*n* = 141	*n* = 39	*n* = 343	
ER-positive^a^ (%)	51.3^b^	38.0^b^	60.0^b^	55.8^b^	
Among women with ER-positive^a^ tumor					
% of tumor cell nuclei positively stained for ER	64.8 (24.9)	67.2 (25.9)	58.3 (26.9)	65.0 (24.3)	0.35
ER H-score^c^	100.9 (52.8)	105.5 (55.2)	90.0 (58.4)	101.0 (51.5)	0.50
ER/PR/HER2 score^d^	–60.3 (41.8)	–54.3 (42.1)	–54.8 (37.4)	–62.7 (42.1)	0.35

Data presented as mean (standard deviation). ER, estrogen receptor; PR, progesterone receptor; HER2, human epidermal growth factor receptor 2.

^a^ER-positive, ≥1% positively stained tumor cell nuclei.

^b^The distribution of age at diagnosis in each subgroup was adjusted according to the distribution of age at diagnosis among the 1,204 participants (35 to 39, 40 to 44, 45 to 49, 50 to 54, 55 to 59, or 60 to 64 years).

^c^ER Histo (H)-score = (% of positively stained tumor cell nuclei at weak intensity category × 1) + (% of positively stained tumor cell nuclei at intermediate intensity category × 2) + (% of positively stained tumor cell nuclei at strong intensity category × 3).

^d^ER/PR/HER2 score = 94.7 × (–0.100 ER_10_ – 0.079 PR_10_ + 0.586 HER2).

Among all women with ER-positive breast cancer, the mean percentage of tumor cell nuclei positively stained for ER was 64.7%, the mean ER H-score was 100.8, and the mean ER/PR/HER2 score was –64.8. The percentage of tumor cell nuclei positively stained for ER and the ER H-score were, on average, lower among those who died from breast cancer than among those in other outcome groups in white women (both *P*_F-test_ ≤0.03), but not in black women (both *P*_F-test_ ≥0.35). Women who died from breast cancer had a higher ER/PR/HER2 score than those in the other outcome groups in white women (mean ER/PR/HER2 score: –46.1, –78.8, –70.8 for three outcome groups, respectively; *P*_F-test_ <0.0001), but not in black women (mean ER/PR/HER2 score: –54.3, –54.8, –62.7 for three outcome groups, respectively; *P*_F-test_ = 0.35).

### Percentage of tumor cell nuclei positively stained for ER and breast cancer-specific mortality risk

**Figure 2:**
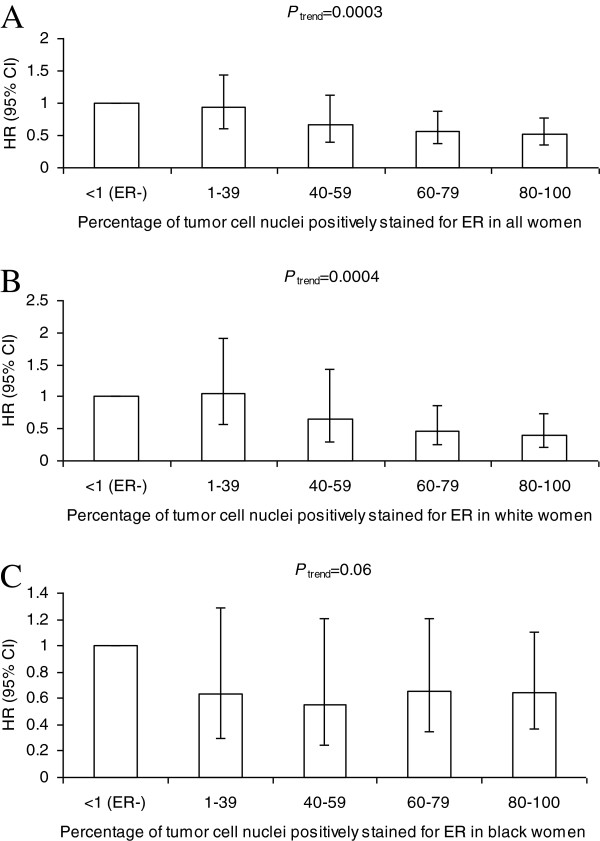
**Percentage of tumor cell nuclei positively stained for estrogen receptor and breast cancer-specific mortality risk.** Adjusted hazard ratio (HR) estimates (95% confidence intervals (CIs)) of breast cancer-specific mortality associated with the percentage of tumor cell nuclei positively stained for estrogen receptor (ER) in all women **(A)**, in white women **(B)**, and in black women **(C)** with invasive breast cancer.

After controlling for age at diagnosis, race, study site, tumor stage, and histologic grade, we found that ER-positivity was associated with reduced risk of breast cancer-specific mortality (Table 2, HR = 0.64, 95% CI = 0.48 to 0.85). Further, the percentage of tumor cell nuclei positively stained for ER was inversely associated with breast cancer-specific mortality risk (*P*_trend_ = 0.0003, Figure 2A). Risk estimates for breast cancer-specific mortality decreased 6% (95% CI = 3 to 9%) for each 10% increase in the percentage of tumor cell nuclei positively stained for ER.

**Table 2:** Breast cancer-specific mortality associated with percentage of tumor cell nuclei positively stained for estrogen receptor

**% of cells staining**	**Number of women**	**Number of deaths**	**Adjusted HR (95% CI)**	
			**Model 1**^**a**^	**Model 2**^**b**^
**Overall**				
<1 (ER-negative)	506	157	Referent	Referent
≥1 (ER-positive)	698	115	0.53 (0.41 to 0.68)	0.64 (0.48 to 0.85)
1 to 39	117	27	0.81 (0.53 to 1.24)	0.93 (0.60 to 1.43)
40 to 59	118	18	0.55 (0.34 to 0.91)	0.66 (0.39 to 1.12)
60 to 79	193	31	0.50 (0.33 to 0.74)	0.57 (0.38 to 0.88)
80 to 100	270	39	0.43 (0.30 to 0.62)	0.52 (0.35 to 0.78)
Trend			*P*_trend_ <0.0001	*P*_trend_ = 0.0003
Trend in women with ER-positive tumors only			*P*_trend_ = 0.004	*P*_trend_ = 0.006
Per 10% increase			0.92 (0.89 to 0.95)	0.94 (0.91 to 0.97)
Per 10% increase in women with ER-positive tumors only			0.91 (0.84 to 0.99)	0.91 (0.84 to 0.99)
**White women**				
<1 (ER-negative)	251	69	Referent	Referent
≥1 (ER-positive)	430	62	0.42 (0.29 to 0.61)	0.56 (0.36 to 0.87)
1 to 39	75	18	0.81 (0.46 to 1.43)	1.04 (0.57 to 1.91)
40 to 59	66	10	0.46 (0.22 to 0.94)	0.65 (0.30 to 1.43)
60 to 79	123	17	0.37 (0.21 to 0.66)	0.47 (0.25 to 0.86)
80 to 100	166	17	0.28 (0.16 to 0.50)	0.39 (0.21 to 0.73)
Trend			*P*_trend_ <0.0001	*P*_trend_ = 0.0004
Trend in women with ER-positive tumors only			*P*_trend_ = 0.001	*P*_trend_ = 0.002
Per 10% increase			0.88 (0.84 to 0.93)	0.91 (0.86 to 0.96)
Per 10% increase in women with ER-positive tumors only			0.85 (0.76 to 0.96)	0.86 (0.76 to 0.96)
**Black women**				
<1 (ER-negative)	255	88	Referent	Referent
≥1 (ER-positive)	268	53	0.56 (0.39 to 0.81)	0.62 (0.42 to 0.94)
1 to 39	42	9	0.56 (0.28 to 1.15)	0.63 (0.30 to 1.29)
40 to 59	52	8	0.50 (0.24 to 1.06)	0.55 (0.25 to 1.21)
60 to 79	70	14	0.60 (0.33 to 1.08)	0.65 (0.35 to 1.21)
80 to 100	104	22	0.56 (0.34 to 0.93)	0.64 (0.37 to 1.10)
Trend			*P*_trend_ = 0.007	*P*_trend_ = 0.06
Trend in women with ER-positive tumors only			*P*_trend_ = 0.54	*P*_trend_ = 0.67
Per 10% increase			0.94 (0.90 to 0.98)	0.95 (0.91 to 1.00)
Per 10% increase in women with ER-positive tumors only			0.98 (0.85 to 1.12)	1.00 (0.87 to 1.14)
**Homogeneity between white women and black women**				
ER-positive versus ER-negative			*P*_homogeneity_ = 0.56	*P*_homogeneity_ = 0.55
Trend			*P*_homogeneity_ = 0.13	*P*_homogeneity_ = 0.12
Trend in women with ER-positive tumors only			*P*_homogeneity_ = 0.05	*P*_homogeneity_ = 0.02
Per 10% increase			*P*_homogeneity_ = 0.16	*P*_homogeneity_ = 0.15
Per 10% increase in women with ER-positive tumors only			*P*_homogeneity_ = 0.06	*P*_homogeneity_ = 0.02

CI, confidence interval; ER, estrogen receptor; HR, hazard ratio.

^a^Multivariable Cox proportional hazards regression models using age (in days) as the time metric stratified by age (in years) and adjusted for race (white, black), study site (Detroit, Los Angeles), tumor stage (localized, non-localized).

^b^Additionally adjusted for histologic grade (low, intermediate, high).

Analyses by race showed that ER-positivity was associated with reduced risk of breast cancer-specific mortality in whites and blacks (test for homogeneity of HRs for ER-positive vs. ER-negative from Model 2: *P* = 0.55). However, the statistically significant inverse association between the percentage of tumor cell nuclei positively stained for ER and breast cancer-specific mortality risk was observed in white women (*P*_trend_ = 0.0004; Figure 2B), but not in black women (*P*_trend_ = 0.06; Figure 2C). The trends in risk with increasing percentages of tumor cell nuclei positively stained for ER for whites and blacks did not differ statistically when both ER-positive and ER-negative women were studied (test for homogeneity of trends from Model 2: *P* = 0.12 for trends using the categorical variable representation and *P* = 0.15 for trends representing each 10% increase in tumor cell nuclei positively stained for ER), but were statistically different in the analyses restricted to women with ER-positive breast cancer (test for homogeneity of trends between white women and black women from Model 2: *P* = 0.02 for both trends using the categorical variable representation and trends representing each 10% increase in tumor cell nuclei positively stained for ER).

### Estrogen receptor Histo-score and breast cancer-specific mortality risk

**Figure 3:**
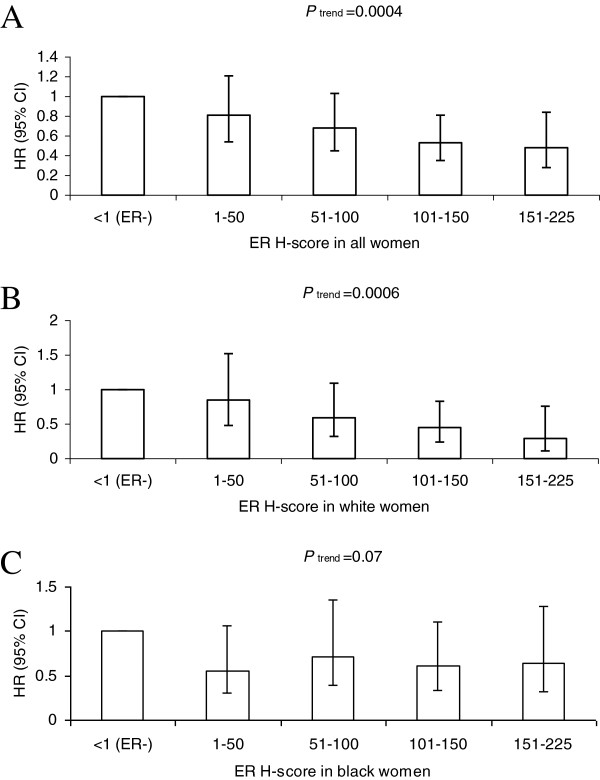
**Estrogen receptor Histo-score and breast cancer-specific mortality risk.** Adjusted hazard ratio (HR) estimates (95% confidence intervals (CIs)) of breast cancer-specific mortality associated with the estrogen receptor (ER) Histo (H)-score in all women **(A)**, in white women **(B)**, and in black women **(C)** with invasive breast cancer.

After controlling for age at diagnosis, race, study site, tumor stage, and histologic grade, we found that ER H-scores were inversely associated with breast cancer-specific mortality risk (Table 3, *P*_trend_ = 0.0004; Figure 3A). Risk estimates for breast cancer-specific mortality decreased 8% (95% CI = 3 to 12%) for each 20 unit increase in ER H-score.

**Table 3:** Breast cancer-specific mortality associated with the estrogen receptor Histo-score

**ER H-score**^**a**^	**Number of women**	**Number of deaths**	**Adjusted HR (95% CI)**	
			**Model 1**^**b**^	**Model 2**^**c**^
**Overall**				
<1 (ER-negative)	506	157	Referent	Referent
1 to 50	173	34	0.70 (0.48 to 1.03)	0.81 (0.54 to 1.21)
51 to 100	173	31	0.58 (0.39 to 0.87)	0.68 (0.45 to 1.03)
101 to 150	226	33	0.45 (0.31 to 0.67)	0.53 (0.35 to 0.81)
151 to 225	126	17	0.39 (0.23 to 0.65)	0.48 (0.28 to 0.84)
Trend			*P*_trend_ <0.0001	*P*_trend_ = 0.0004
Trend in women with ER-positive tumors only			*P*_trend_ = 0.008	*P*_trend_ = 0.02
Per 20 unit increase			0.90 (0.86 to 0.94)	0.92 (0.88 to 0.97)
Per 20 unit increase in women with ER-positive tumors only			0.90 (0.83 to 0.98)	0.90 (0.84 to 0.99)
**White women**				
<1 (ER-negative)	251	69	Referent	Referent
1 to 50	107	22	0.66 (0.39 to 1.11)	0.85 (0.48 to 1.52)
51 to 100	104	17	0.46 (0.26 to 0.82)	0.59 (0.32 to 1.09)
101 to 150	141	18	0.34 (0.19 to 0.58)	0.45 (0.24 to 0.83)
151 to 225	78	5	0.20 (0.08 to 0.52)	0.29 (0.11 to 0.76)
Trend			*P*_trend_ <0.0001	*P*_trend_ = 0.0006
Trend in women with ER-positive tumors only			*P*_trend_ = 0.008	*P*_trend_ = 0.01
Per 20 unit increase			0.69 (0.59 to 0.79)	0.75 (0.64 to 0.88)
Per 20 unit increase in women with ER-positive tumors only			0.66 (0.49 to 0.90)	0.68 (0.50 to 0.92)
**Black women**				
<1 (ER-negative)	255	88	Referent	Referent
1 to 50	66	12	0.51 (0.27 to 0.94)	0.56 (0.30 to 1.06)
51 to 100	69	14	0.66 (0.36 to 1.19)	0.72 (0.39 to 1.35)
101 to 150	85	15	0.55 (0.31 to 0.99)	0.61 (0.33 to 1.11)
151 to 225	48	12	0.54 (0.28 to 1.05)	0.64 (0.32 to 1.28)
Trend			*P*_trend_ = 0.008	*P*_trend_ = 0.07
Trend in women with ER-positive tumors only			*P*_trend_ = 0.41	*P*_trend_ = 0.59
Per 20 unit increase			0.93 (0.88 to 0.98)	0.95 (0.89 to 1.01)
Per 20 unit increase in women with ER-positive tumors only			0.96 (0.85 to 1.09)	0.97 (0.86 to 1.10)
**Homogeneity between white women and black women**				
Trend			*P*_homogeneity_ = 0.10	*P*_homogeneity_ = 0.09
Trend in women with ER-positive tumors only			*P*_homogeneity_ = 0.05	*P*_homogeneity_ = 0.02
Per 20 unit increase			*P*_homogeneity_ = 0.08	*P*_homogeneity_ = 0.08
Per 20 unit increase in women with ER-positive tumors only			*P*_homogeneity_ = 0.05	*P*_homogeneity_ = 0.02

CI, confidence interval; ER, estrogen receptor; HR, hazard ratio.

^a^ER Histo-score = (% of cells stained at weak intensity category × 1) + (% of cells stained at intermediate intensity category × 2) + (% of cells stained at strong intensity category × 3).

^b^Multivariable Cox proportional hazards regression models using age (in days) as the time metric stratified by age (in years) and adjusted for race (white, black), study site (Detroit, Los Angeles), tumor stage (localized, non-localized).

^c^Additionally adjusted for histologic grade (low, intermediate, high).

Analyses by race showed that the statistically significant inverse association for white women (*P*_trend_ = 0.0006; Figure 3B), but not for black women (*P*_trend_ = 0.07; Figure 3C). The difference between whites and blacks was not statistically significant when ER-positive and ER-negative women were included (test for homogeneity of trends from Model 2: *P* = 0.09 for trends using the categorical variable representation and *P* = 0.08 for trends representing each 20 unit increase in ER H-score), but it was statistically significant in analyses restricted to women with ER-positive breast cancer (test for homogeneity of trends between white women and black women from Model 2: *P* = 0.02 for both trends using categorical variable representation and the trends representing each 20 unit increase in ER H-score).

### ER/PR/HER2 score and breast cancer-specific mortality risk

**Figure 4:**
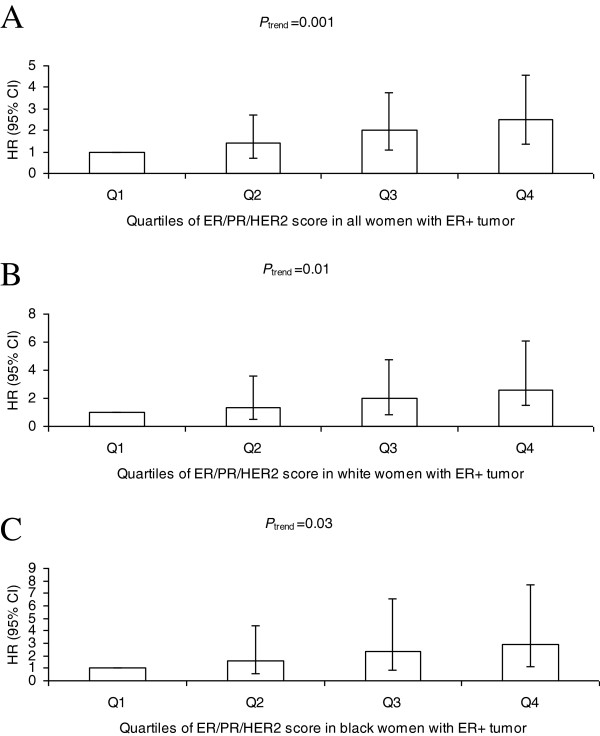
**ER/PR/HER2 score and breast cancer-specific mortality risk in women diagnosed with estrogen receptor-positive breast cancer.** Adjusted hazard ratio (HR) estimates (95% confidence intervals (CIs)) of breast cancer-specific mortality associated with the estrogen receptor/progesterone receptor/ human epidermal growth factor receptor 2 (ER/PR/HER2) score in all women **(A)**, in white women **(B)**, and in black women **(C)** with ER-positive invasive breast cancer.

After controlling for age at diagnosis, race, study site, tumor stage, and histologic grade, no statistically significant association between the ER/PR/HER2 score and breast cancer-specific mortality risk was observed in women with ER-negative breast cancer (Table 4). However, in women with ER-positive tumors, the ER/PR/HER2 score was positively associated with breast cancer-specific mortality risk (*P*_trend_ = 0.001; Figure 4A); the HR for breast cancer death was 2.48 (95% CI = 1.36 to 4.55) for those in the highest quartile of the ER/PR/HER2 score compared with those in the lowest quartile. The positive association was observed for both white women (*P*_trend_ = 0.01; Figure 4B) and black women (*P*_trend_ = 0.03; Figure 4C) with ER-positive tumor.

**Table 4:** Breast cancer-specific mortality associated with the ER/PR/HER2 score

**ER/PR/HER2 score**^**a**^	**Number of women**	**Number of deaths**	**Adjusted HR (95% CI)**	
			**Model 1**^**b**^	**Model 2**^**c**^
**Women with ER-negative invasive breast cancer**				
Overall				
< 0	60	15	Referent	Referent
0	337	103	1.32 (0.76 to 2.32)	1.30 (0.74 to 2.28)
> 0	109	39	1.43 (0.77 to 2.69)	1.43 (0.76 to 2.67)
Trend			*P*_trend_ = 0.30	*P*_trend_ = 0.29
White women				
< 0	35	9	Referent	Referent
0	162	46	1.20 (0.55 to 2.62)	1.09 (0.48 to 2.45)
> 0	54	14	1.16 (0.46 to 2.90)	1.09 (0.43 to 2.76)
Trend			*P*_trend_ = 0.79	*P*_trend_ = 0.88
Black women				
< 0	25	6	Referent	Referent
0	175	57	1.46 (0.59 to 3.61)	1.47 (0.60 to 3.64)
> 0	55	25	1.57 (0.61 to 4.06)	1.55 (0.60 to 4.03)
Trend			*P*_trend_ = 0.42	*P*_trend_ = 0.45
**Homogeneity of trends between white women and black women**			*P*_homogeneity_ = 0.36	*P*_homogeneity_ = 0.32
**Women with ER-positive invasive breast cancer**				
Quartiles of ER/PR/HER2 score^d^				
Overall				
Q1	175	18	Referent	Referent
Q2	177	22	1.27 (0.65 to 2.45)	1.39 (0.71 to 2.70)
Q3	172	33	2.04 (1.10 to 3.81)	2.00 (1.07 to 3.76)
Q4	174	42	2.71 (1.48 to 4.95)	2.48 (1.36 to 4.55)
Trend			*P*_trend_ = 0.0002	*P*_trend_ = 0.001
White women				
Q1	118	9	Referent	Referent
Q2	105	10	1.41 (0.53 to 3.74)	1.37 (0.52 to 3.62)
Q3	113	20	2.03 (0.84 to 4.92)	1.98 (0.82 to 4.78)
Q4	94	23	2.77 (1.19 to 6.45)	2.62 (1.12 to 6.11)
Trend			*P*_trend_ = 0.01	*P*_trend_ = 0.01
Black women				
Q1	57	9	Referent	Referent
Q2	72	12	1.32 (0.50 to 3.51)	1.60 (0.58 to 4.45)
Q3	59	13	2.34 (0.85 to 6.42)	2.34 (0.83 to 6.55)
Q4	80	19	3.43 (1.33 to 8.84)	2.88 (1.08 to 7.67)
Trend			*P*_trend_ = 0.006	*P*_trend_ = 0.03
**Homogeneity of trends between white women and black women**			*P*_homogeneity_ = 0.56	*P*_homogeneity_ = 0.32

CI, confidence interval; ER, estrogen receptor; HER2, human epidermal growth factor receptor 2; HR, hazard ratio; PR, progesterone receptor.

^a^ER/PR/HER2 score = 94.7 × (–0.100 ER_10_ – 0.079 PR_10_ + 0.586 HER2); the range of ER/PR/HER2 scores was –71.07 to 55.49 in women with ER-negative tumor and –141.10 to 53.92 in women with ER-positive tumor.

^b^Multivariable Cox proportional hazards regression models using age (in days) as the time metric stratified by age (in years) and adjusted for race (white, black), study site (Detroit, Los Angeles), tumor stage (localized, non-localized).

^c^Additionally adjusted for histologic grade (low, intermediate, high); low and intermediate were combined into a single category in analyses for women with ER-negative tumors due to a small number of women with a low grade.

^d^Quartiles of ER/PR/HER2 scores in all women with ER-positive tumors were Q1 (–141.10 to –97.33), Q2 (–97.32 to –68.98), Q3 (–68.97 to –36.66), and Q4 (–36.65 to 53.92).

## Discussion

Our data showed that both the percentage of tumor cell nuclei positively stained for ER and the ER H-score were inversely associated with breast cancer-specific mortality risk in all women and among those with ER-positive breast cancer. The ER/PR/HER2 score was positively associated with breast cancer-specific mortality risk in women with ER-positive tumors. Analyses by race revealed that ER-positivity was associated with reduced risk of breast cancer-specific mortality in white women and black women. Both quantitative measures for ER alone provided additional discrimination in breast cancer-specific mortality risk for white women but not black women with ER-positive tumors, while the ER/PR/HER2 score provided the additional discrimination for both white women and black women with ER-positive tumors.

The association between the quantity of ER and patients’ mortality risk or survival after diagnosis has been examined in six studies, three that used the DCC assay [23-25] and three that used the IHC assay [21,26,27]. The largest study using the DCC assay was conducted among 1,184 patients diagnosed with breast cancer between 1975 and 1981 who had a median follow-up of 5 years. This study found that increased concentration of ER in tissue homogenates was associated with increased probability of breast cancer-free survival, and this persisted after additional stratification of the data by lymph node status, tumor stage, and menopausal status [25].

Among the studies that used IHC, one reported that the relative risk for all-cause mortality, adjusted for tumor size and the percentage of nuclei stained for proliferation-related antigen, was 0.65 (95% CI = 0.30 to 1.43) when comparing patients with >10% with those with ≤10% of ER positively immunostained tumor cell nuclei and was 0.44 (95% CI = 0.21 to 0.93) when comparing >30% with those with ≤30% of ER [26]. This study was a small study with 180 primary breast cancer patients, who had between 4 and 7 years of clinical follow-up. Among 205 patients with ER-positive metastatic breast cancer (defined by DCC) who were followed for a median of 9 years, the percentage of ER positively immunostained tumor cell nuclei was positively associated with the cumulative probability of survival [27]. Another study, which included 563 postmenopausal patients with stage I or stage II breast cancer, examined the relationships of the cumulative probability of survival with H-score and with the percentage of tumor cell nuclei positive for ER [21]. These patients all received adjuvant tamoxifen, but no chemotherapy following surgical resection of histologically confirmed ER-positive breast cancer. The investigators reported that the cumulative probability of survival was positively associated with ER H-score (10-year survival: 41%, 71%, 67%, and 84% for H-scores of >0 to 50, 51 to 100, 101 to 200, and >200, respectively), but found no association with the percentage of tumor cells that had ER-positive nuclei (10-year survival: 65.5%, 43.4%, and 70.9% for the percentage of cells staining for ER of >0 to <34%, 34 to 67%, and >67%, respectively). All together, the previous studies, most of which were hospital based, provide some evidence that a higher level of ER expression in tumor tissue is associated with decreased mortality risk or better survival after breast cancer diagnosis. Our study using IHC, based on a larger population-based sample, found that the multivariable-adjusted relative hazard for breast cancer-specific mortality decreased with increasing values of the two quantitative measures of ER alone.

The observed inverse association of breast cancer-specific mortality risk with increased quantity of ER may be due to the positive association between ER quantity and response to endocrine therapy [24,28]. Clinical trials have found that higher levels of ER expression in tumors were associated with a lower relative risk of recurrence [29,30]. Additionally, the inverse association may be related to the biological characteristics of breast cancers. Supporting evidence indicates that increasing recurrence-free survival is associated with higher ER expression even in patients who did not receive any adjuvant treatment [25]. Early studies reported an inverse association between the proliferation rate, determined by the thymidine labeling index, and ER content [31,32]. A higher thymidine labeling index was associated with unfavorable outcomes such as early relapse and shorter survival time [33].

It is unclear why the inverse associations between two quantitative measures of ER alone and breast cancer-specific mortality were observed in white women but not in black women with ER-positive breast cancer. It is important to note that the number of black women with ER-positive breast cancer is relatively small compared with white women with ER-positive breast cancer in our study sample. Further, if black women were less likely than white women to receive optimal treatment it is possible that we would not observe a decreasing risk of death with the percentage of tumor cell nuclei positively stained for ER or the ER H-score. Prior studies have suggested that black women may receive less optimal treatment than white women [34-37]. However, we were unable to assess treatment differences because treatment information was not collected.

Cuzick and colleagues reported that the combined score of ER H-score with PR/HER2/Ki-67 had prognostic value for the distant recurrence in postmenopausal women who were diagnosed with ER-positive breast cancer [11]. Similarly, our data, using an adaptation of Cuzick’s score, showed that the ER/PR/HER2 score had prognostic value for breast cancer-specific mortality for both white women and black women with ER-positive tumors. Our data suggest that the weighted combination of ER/PR/HER2 score provides better prognostic discrimination for breast cancer-specific mortality risk among women with ER-positive tumors than that observed for quantitative measures of ER alone. Although Cuzick and colleagues also found that Ki-67 provided prognostic information in addition to ER/PR/HER2 for the risk of the distance recurrence of breast cancer [11], we unfortunately did not assay Ki-67. Therefore, it would be valuable to assess the prognostic value of Ki-67 for breast cancer-specific mortality risk in the future studies.

## Conclusions

Our data indicate that quantitative IHC measures of ER, especially the ER/PR/HER2 score, are more precise predictors for breast cancer-specific mortality risk than a simple determination of ER positivity.

## Abbreviations

CARE: Contraceptive and reproductive experiences; CI: Confidence interval; DCC: Dextran-coated charcoal; ER: Estrogen receptor; HER2: Human epidermal growth factor receptor 2; HR: Hazard ratio; H-score: Histo-score; IHC: Immunohistochemistry; LA: Los Angeles; PR: Progesterone receptor; SEER: Surveillance epidemiology and end results.

## Competing interests

The authors declare that they have no competing interests.

## Authors’ contributions

PAM, BLS, LKW, KEM, DMD, and LB conceived of and designed the Women’s CARE Study. PAM, BLS, LKW, KEM, DMD, SGF, JAM, RTB, MSS, JS-H, and LB supervised or participated in the data collection and assembly of data of the Women’s CARE Study. MSS, RTB and MFP helped to interpret medical questions during the conduct of the Women’s CARE Study. MFP and LB conceived of and designed the pathology substudy. MFP conducted the assessment of biomarkers. HM conceived of the specific analytic questions investigated in this paper. HM and YL conducted data analyses under LB’s supervision. HM interpreted the results and drafted the manuscript with LB’s input. All authors participated in the critical revision of the manuscript and read and approved the final version.
